# Optimization of the RNA extraction method for transcriptome studies of *Salmonella *inoculated on commercial raw chicken breast samples

**DOI:** 10.1186/1756-0500-4-60

**Published:** 2011-03-11

**Authors:** Sujata A Sirsat, Arunachalam Muthaiyan, Steven C Ricke

**Affiliations:** 1 Dept. of Poultry Science, University of Arkansas, Fayetteville, AR 72701, USA; 2 Center for Food Safety-IFSE and Dept. of Food Science, University of Arkansas, 2650 N. Young Ave, Fayetteville AR 72704-5690, USA

## Abstract

**Background:**

There has been increased interest in the study of molecular survival mechanisms expressed by foodborne pathogens present on food surfaces. Determining genomic responses of these pathogens to antimicrobials is of particular interest since this helps to understand antimicrobial effects at the molecular level. Assessment of bacterial gene expression by transcriptomic analysis in response to these antimicrobials would aid prediction of the phenotypic behavior of the bacteria in the presence of antimicrobials. However, before transcriptional profiling approaches can be implemented routinely, it is important to develop an optimal method to consistently recover pathogens from the food surface and ensure optimal quality RNA so that the corresponding gene expression analysis represents the current response of the organism. Another consideration is to confirm that there is no interference from the "background" food or meat matrix that could mask the bacterial response.

**Findings:**

Our study involved developing a food model system using chicken breast meat inoculated with mid-log *Salmonella *cells. First, we tested the optimum number of *Salmonella *cells required on the poultry meat in order to extract high quality RNA. This was analyzed by inoculating 10-fold dilutions of *Salmonella *on the chicken samples followed by RNA extraction. Secondly, we tested the effect of two different bacterial cell recovery solutions namely 0.1% peptone water and RNAprotect (Qiagen Inc.) on the RNA yield and purity. In addition, we compared the efficiency of sonication and bead beater methods to break the cells for RNA extraction. To check chicken nucleic acid interference on downstream *Salmonella *microarray experiments both chicken and *Salmonella *cDNA labeled with different fluorescent dyes were mixed together and hybridized on a single *Salmonella *array. Results of this experiment did not show any cross-hybridization signal from the chicken nucleic acids. In addition, we demonstrated the application of this method in a meat model transcriptional profiling experiment by studying the transcriptomic response of *Salmonella *inoculated on chicken meat and exposed to d-limonene. We successfully applied our method in this experiment to recover the bacterial cells from the meat matrix and to extract the RNA. We obtained high yield and pure RNA. Subsequently, the RNA was used for downstream transcriptional profiling studies using microarrays and over 600 differentially regulated genes were identified.

**Conclusions:**

Our result showed that 8 log cfu/g of *Salmonella *is ideal to obtain optimal RNA amount and purity. Our results demonstrated that RNAprotect yielded higher RNA amounts (approximately 10 to 30 fold) when compared to 0.1% peptone water. The differences between the RNAprotect and 0.1% peptone samples were significant at a p-value of 0.03 for the bead beater method and 0.0005 for the sonication method, respectively. The microarray experiment demonstrated that the chicken samples do not interfere with the hybridization of *Salmonella *cDNA on the array slide. Hence, the background chicken RNA will not interfere with the microarray analysis when poultry meat models are used. Finally, we successfully demonstrated the application of the poultry meat model proposed in this study by conducting transcriptional profiling analysis of *Salmonella *inoculated on the poultry. Results of this study proved that this method has the potential to be employed in other meat model studies.

## Background

Disease caused by foodborne pathogens contributes to serious public health concerns [1]. The Food Safety and Inspection Services (FSIS) have stated that *Salmonella *is the most common cause of foodborne illness among enteric pathogens [2]. The World Health Organization (WHO) has reported that salmonellosis is reemerging as an important infectious disease worldwide [3]. According to the USDA-FSIS reports, presence of *Salmonella *due to fecal contamination of carcasses is a major issue for the poultry industry. Additionally, the USDA-FSIS report established a "*Salmonella *verification program with a goal of having 90% of the poultry houses meeting new standards" [4]. *Salmonella *is one of the leading foodborne pathogens potentially present on the surface of poultry. Several hurdles are used by the poultry and meat industry to combat these foodborne pathogens and eliminate their presence on carcasses. Chemical washes such as chlorine, organic acids, cetylpyridinium chloride, and trisodium phosphate are commonly used in the United States but are prohibited in some countries belonging to the European Union (EU) [5-7].

It is important to understand the physiological status of the pathogens stressed by these antimicrobial treatments as well as their genomic and proteomic responses. This will enable researchers to identify the best possible combinations of antimicrobial treatment that does not lead to cross-protection responses by the pathogen [8]. Studying the gene or protein expression of foodborne pathogens present on food surfaces such as chicken enables a better understanding of genomic expression in the actual food matrix. For instance, a previous study followed the expression of the staphylococcal enterotoxin A (SEA) gene of *Staphylococcus aureus *on the surface of pork products over time [9].

To study the gene expression of pathogens on the surface of chicken carcass, recovery of cells and their corresponding purity of RNA is crucial. Therefore, in this study we compared RNA extraction methods for pathogen-poultry surface transcriptome studies. In order to develop this standard method, two different bacterial cell recovery solutions (0.1% peptone water and RNAprotect solution) and two different cell lysing methods (sonication and bead beater), were used to recover the *Salmonella *from the chicken meat surface and perform the subsequent RNA extractions. In addition, the effect of "the background meat matrix" cross-contamination in the downstream expression profiling study was evaluated by hybridizing the chicken tissue RNA and *Salmonella *RNA to the *Salmonella *genomic microarrays. Subsequently, to test this methodology we applied this method to study the transcriptional response of *Salmonella *inoculated on poultry meat and exposed to d-limonene antimicrobial treatment. In this experiment *Salmonella *cells were recovered from the poultry meat treated with d-limonene (test) and sterile water (control). The cell recovery and RNA extraction was carried out by the method described in this paper and the RNA was used for downstream transcriptomic analysis using microarrays.

## Methods

### Bacterial strains, culture media, and growth conditions

*Salmonella enterica *serovar Typhimurium ATCC 14028 from the culture collection of the Center for Food Safety, University of Arkansas was used for this study. Stock cultures of the strain were held in Luria Bertani broth (LB) and glycerol at -80°C. The cultures were grown overnight in LB at 37°C and transferred twice before use in the experiment.

### Chemicals

The citrus oil, d-limonene, was obtained from Firmenich Citrus Center, Safety Harbor, FL, and stored at 4°C as per the manufacturer's recommendation.

### Chicken inoculation and treatments

**Figure 1:**
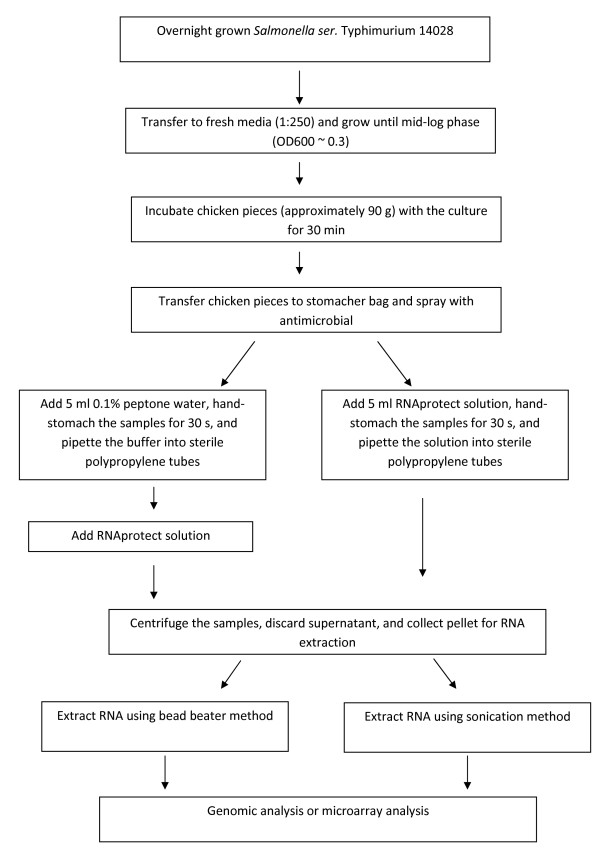
**Method used for bacterial recovery and RNA extraction from chicken breast samples**.

The outline of the overall method is shown in Figure 1. Overnight cultures were transferred to fresh LB media in a ratio of 1:250 and grown until bacteria reached mid-log phase. The chicken pieces (approximately 25 g) were inoculated with approximately 8, 7, 6, and 5 log cfu/g bacterial cells at mid-log phase in a sterile flask and incubated for 30 min at room temperature. The pieces were subsequently transferred to sterile stomacher bags using sterile forceps. Following this, the samples were stomached by hand for 30 sec and the cells were recovered using 5 ml of either 0.1% peptone water or RNAprotect solution (Qiagen Inc., Valencia, CA). The cells were collected in 50 ml polypropylene tubes and centrifuged at 10,000 rpm for 10 minutes at 4°C (Beckman J2-21, Brea, CA). The supernatant was discarded and the pellets were used for RNA isolation.

### Chicken tissue collection for RNA extraction

Chicken breast was purchased at retail stores, cut into pieces in a sterile environment, and stored in sterile stomacher bags. Five ml of RNAprotect solution was added directly to the stomacher bag and stomached manually. The liquid portion was recovered and centrifuged in polypropylene tubes. The pellets were stored at -80°C overnight and used for subsequent RNA isolations.

### d-limonene treatments

The chicken samples were inoculated with mid-log (OD_600 _approximately 0.4) *Salmonella *for 30 min. The pieces were placed in sterile stomacher bags and sprayed with either d-limonene (test) or sterile water (control) and incubated for 15 min. Five ml RNAprotect was added to the stomacher bags and stomached by hand for 30 sec. The liquid portion was transferred by pipette to 50 ml polypropylene tubes and centrifuged at 10,000 rpm for 10 minutes at 4°C (Beckman J2-21, Brea, CA). The supernatant was discarded and pellets were used for RNA isolation.

### RNA isolation, quantification, and storage conditions

The RNeasy mini kit (Qiagen Inc.) was used for RNA isolation with some modifications described in the next section. One ml of TRIzol (Invitrogen, Carlsbad, CA) was added to the bacterial or chicken tissue sample and vortexed for 40 sec. The samples were either subjected to sonication or to a bead beater in order to break the cells. For the sonication method, the sample was transferred to a glass sonicator tube and sonicated three times at 1 min intervals (Misonix Inc., Farmingdale, NY). For the bead beater process, the samples were transferred to FastPrep blue tubes containing glass beads (MP Biomedicals, Solon, OH). The cells were broken in FastPrep for 40 sec at a bead beating frequency speed setting of number 6. Following this, 200 μl of chloroform was added and then centrifuged at 4°C for 15 min at 12,000 rpm (VWR Galaxy 16, West Chester, PA). The upper phase containing nucleic acid was collected and transferred to an RNeasy mini kit column and further purification steps were conducted according to kit instructions. In addition, the RNA was subjected to DNase treatment (Qiagen Inc.), to remove any DNA residues that could affect the downstream reaction. The total RNA quantity and purity ratios were calculated using a Nanodrop 8000 (Thermo Scientific, Wilmington, DE). The RNA was stored at -80°C and used for downstream molecular analysis.

### Microarray analysis

cDNA was synthesized from the purified RNA as described by Muthaiyan et al. (2008) [10]. Briefly, 5 μg of the purified RNA from the chicken and *Salmonella *samples were annealed with random hexamers as primers (Invitrogen). The samples were placed in a heating block at 70°C for 5 min and then incubated in ice for 1 min. Following this, SuperScript III reverse transcriptase (Invitrogen) and 0.1 M dithiothreitol 12.5 mM dNTP/aminoallyl-UTP (Ambion, Austin, TX) mix was added and the samples were incubated at 42°C for 18 h. Residual RNA was removed and cDNA was purified with a QIAquick PCR purification kit (Qiagen Inc.). Purified aminoallyl-modified cDNA was subsequently recovered, labeled with Cy3 or Cy5 mono-functional NHS ester cyanogen dyes (Amersham Pharmacia Biotech, Piscataway, NJ), and purified, using a PCR purification kit (Qiagen, Inc.) following the manufacturer's instructions. Purified labeled cDNA were hybridized on *S*. Typhimurium microarray version 5 slides provided by the Pathogen Functional Genomics Resource Center (PFGRC). Hybridized slides were scanned using a GenePix 4000B microarray scanner (Axon Instruments, Union City, CA).

### Statistical analysis

Statistics were done to analyze differences between the two different methods using Microsoft Excel (Microsoft Office 2003). Type 2 t-tests statistics were performed to obtain the p-value and test significant differences among various groups.

## Results and Discussion

There is an increasing need to study the effect of antimicrobial treatments on the transcriptome of the pathogen. Results from these studies enable researchers to elucidate the molecular mechanisms by which foodborne pathogens tolerate and survive on complex food matrices that are subjected to a variety of antimicrobial treatments. Our previous microarray studies have demonstrated the induction of adhesion genes in heat stressed *Salmonella*. These results were further confirmed by the increased adhesion of heat stressed *Salmonella *to Caco-2 cells [11]. Only a few studies reporting the effects of antimicrobial stresses on pathogens inoculated on poultry meat have been documented [12-14]. Transcriptomic studies will aid the understanding of the effect of antimicrobials on pathogens at the molecular level. This will enable researchers to develop efficient antimicrobial intervention methods and potentially avoid cross-protection or the development of resistance mechanisms in the pathogen. Gene expression analysis can potentially indicate the physiological state of the pathogens in response to various hurdles applied in meat processing [15]. Microarrays are a useful tool to carry out transcriptional profiling studies since this technique enables the study of the transcriptome on a global level. In this study we developed a method to study the transcriptomic profile of bacteria from the meat matrix. We standardized the following parameters in order to effectively analyze the bacterial transcriptome: 1. least number of cells required on the chicken sample to obtain RNA for genomic expression analysis, 2. suitable cell recovery solution to obtain high quantity and pure RNA, and 3. ascertain that there was no interference of the background food matrix while performing transcriptional profiling using microarrays. After standardizing this method we successfully demonstrated the significance of this optimized method by administering antimicrobial treatments against *Salmonella *inoculated on a chicken matrix and performing downstream transcriptional profiling analysis.

To test the minimum number of cells required for quantifiable bacterial RNA yield serial dilutions of 8, 7, 6, and 5 log cfu/g mid-log *Salmonella *cells were added to 25 g chicken breast samples. We observed that 8 log cfu/g resulted in a RNA yield of 92.7 ± 6.5 ng/μl with a purity ratio of 2.0 (260/280 and 260/230). The lower dilutions of 7, 6, and 5 log cfu/g mid-log *Salmonella *cells exhibited a lower concentration of RNA with poor purity. Results of this study showed that 8 log cfu/g of bacterial cells were required to obtain both high yield and pure RNA. The RNA yields obtained after inoculating 8 log cfu/g on chicken was more than sufficient for molecular analysis using quantitative real- time reverse transcriptase PCR (qRT-PCR) since the technique requires approximately 100 ng RNA per reaction [16]. However, for microarray analysis, higher RNA yield (approximately 2000 ng/μl) is recommended [17]. For this purpose researchers would need to pool several samples from multiple rinses or from multiple carcasses.

**Figure 2:**
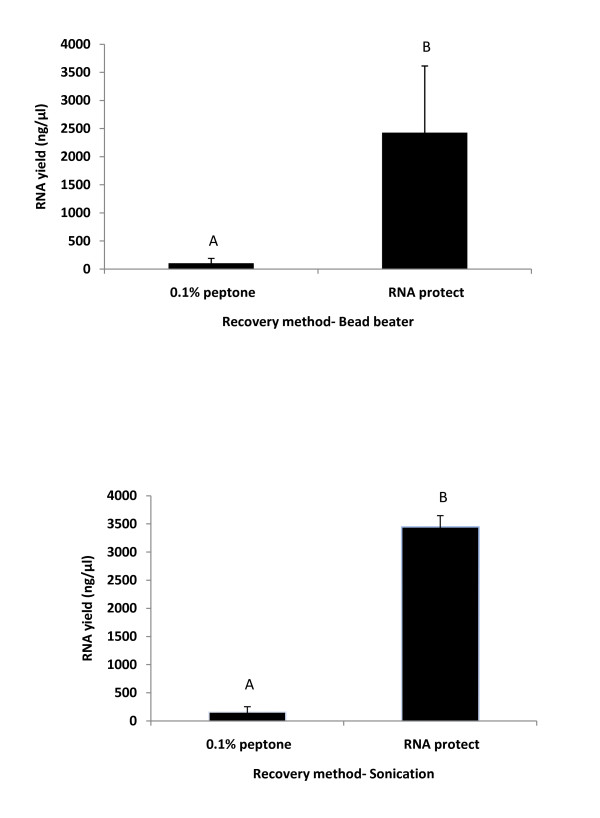
***Salmonella *****RNA yield from twodifferent recovery buffers**. By the: (a) bead beater method [different letters indicate significant difference (p value 0.03).] and (b) sonication method [different letters indicate significant difference (p value 0.0005)].

Several studies involving food models have tested the viability of pathogens on meat samples using recovery buffers such as 0.1% peptone water, saline, or PBS for enumeration of colonies from these bacteria on petri plates [9,12,18-20]. This type of recovery system is effective for cell viability studies. In order to test whether 0.1% peptone water is suitable to recover cells for RNA extraction studies, RNA yields from *Salmonella *cells recovered with 0.1% peptone water and RNAprotect solution using two different methods for cell lysis was tested (Figure 2). RNA yield was highest when the cells were recovered with RNAprotect solution in both the cell lysis methods. Recovery using 0.1% peptone water revealed an approximately 10 to 30 fold reduction in RNA yield compared to recovery using RNAprotect. This could be because RNAprotect preserves the RNA and inhibits the action of RNA degrading nucleases on the RNA [21]. The differences were significant at a p-value of 0.03 for the bead beater method and 0.0005 for the sonication method. No significant differences (p > 0.05) were found between the RNA yields obtained between the bead beater and the sonication method.

RNA purity and concentration are two major factors involved in successful gene expression experiments. RNA purity ensures reliability and reproducibility of downstream analyses and low quantities of recovered RNA may compromise further molecular applications [22]. In our study we found that recovery of bacterial cells using RNAprotect results in higher RNA yield and better RNA purity using both the sonication and bead beater methods. The average 260/280 and 260/230 ratios for the RNA protect and 0.1% peptone water samples processed with both the bead beater and sonication method ranged from 1.9 to 2 and 1.6 to 2 respectively.

**Figure 3:**
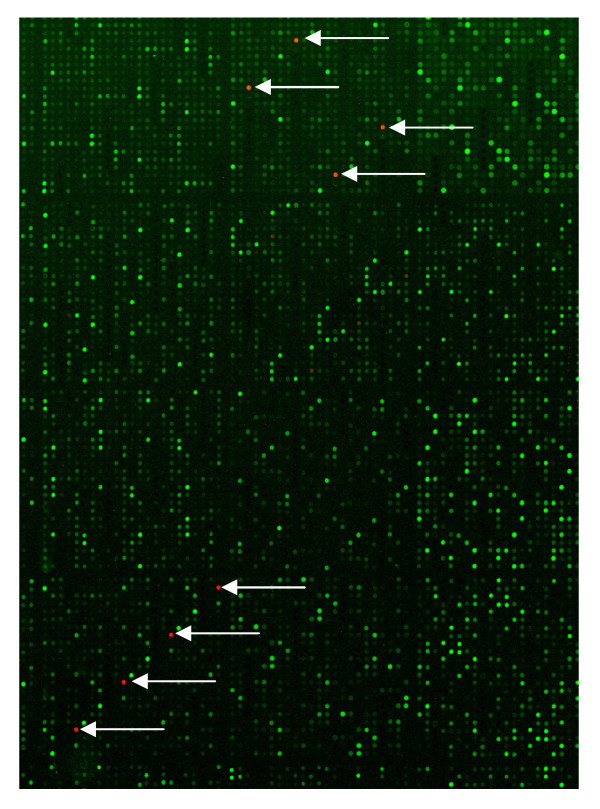
**Comparative hybridization of *Salmonella *and chicken cDNA on *Salmonella *Typhimurium microarray**. Green color spots = *Salmonella *samples; white arrows indicate red color spots = chicken samples.

To ensure that the "background food matrix" (chicken breast) was not interfering with downstream molecular gene expression analysis we performed a microarray analysis using pure culture *Salmonella *and the un-inoculated chicken matrix. We chose to use microarrays for the gene expression studies since this technique has been widely used for studying global gene expression patterns in several organisms [23-28]. This method has several advantages over other techniques including Northern blotting and qRT-PCR since these techniques can analyze only a few genes in one reaction. Conversely, microarrays can analyze the entire genome of the organism in question in a single reaction [29]. In our study the *Salmonella *cDNA sample was labeled with the Cy3 dye and the chicken sample with the Cy5 dye. Additionally, a dye swap control was also performed to avoid dye bias. The Cy3 (cDNA made from *Salmonella *RNA) was the dominant color and very few spots with Cy5 (cDNA made from chicken RNA) hybridization was observed (Figure 3). A scan image revealed only four ORF's that cross hybridized with chicken RNA on the *Salmonella *array. These ORFs belonged to genes *yraL*, *nanK*, genes coding for a transposase, and a cation transport ATPase respectively. In order to identify the cross-hybridized spots a BLAST search was performed using the cross-hybridized *Salmonella *gene sequences against the chicken genome [30]. However, no homology was found between the *Salmonella *and the chicken genome. The hybridization could possibly be a result of very low background microflora that may be present on the chicken sample [31]. Therefore, our study indicates that while using a poultry model for *Salmonella *transcriptional profiling experiments, the interference from chicken nucleic acids is very minimal. Additionally, our results confirm that the entire genome *Salmonella *microarray preferentially binds only with *Salmonella *cDNA.

### Application of our method to study the transcriptional response of *Salmonella *inoculated on meat matrix

In order to examine the practical application of this optimized method we designed an experiment to test the effect of an antimicrobial on the transcriptome of *Salmonella *inoculated on the poultry meat matrix. Previous studies in our laboratory have shown d-limonene, a natural citrus oil, to be inhibitory against several strains of *Salmonella *[32]. Transcriptional studies were carried out by inoculating mid-log (OD_600 _approximately 0.4) *Salmonella *on poultry samples for 30 min as described in the previous sections. The poultry samples were sprayed with d-limonene and *Salmonella *was recovered and used for RNA extractions as described in the previous sections. We obtained 1000 ng/μl of RNA yield with high level purity (260/280 and 260/230 ratios approximately 2). This ratio is generally recommended for downstream molecular analysis [33]. In order to further verify our method, downstream molecular analysis was performed using microarrays to test the gene expression of *Salmonella *exposed to d-limonene. We observed an optimum Cy3 and Cy5 dye labeling of the cDNA synthesized from the RNA extracted from the *Salmonella *cells recovered from the meat matrix. Hybridization signals on the microarray did not display any background signal interference from the chicken RNA. Further analysis of the hybridization signal revealed a total of 359 upregulated and 313 downregulated *Salmonella *genes in response to the d-limonene treatment (Additional file 1: Supplementary table S1). Few studies have been reported on the mode of action of these citrus compounds on foodborne pathogens. Our results demonstrated that several genes from the cell envelope category (62 genes) and cellular processes category (32 genes) were differentially regulated. This may indicate the mode of action of d-limonene is primarily directed toward the membrane of *Salmonella*. Additionally, 160 hypothetical proteins genes were differentially regulated. This result indicates that d-limonene may influence mechanisms within the pathogen that remain to be determined. Overall, these results confirm the use of the proposed optimized poultry model for practical application for researchers who would be interested in conducting transcriptional screening of foodborne pathogens (on complex food matrixes) following the application of antimicrobials (natural or conventional).

## Conclusion

Gene expression analysis of pathogens inoculated on food matrix is crucial to understand the state of the pathogen on a molecular level. This type of analysis is complicated because of background food matrix contamination. Results of our study demonstrated that the meat matrix does not interfere with the microarray analysis of *Salmonella *inoculated on chicken pieces. From a practical standpoint, the methodology demonstrated in this study can be used to recover bacterial cells from the different meat matrices and used in further molecular studies such as microarray and qRT-PCR. The method described in this study could be useful to devise microarray approaches as laboratory tools to screen genes that may be differentially expressed after antimicrobial addition where a large number of bacterial cells would need to be inoculated onto the poultry matrix.

## Competing interests

The authors declare that they have no competing interests.

## Authors' contributions

SAS designed and performed the experiments and drafted the manuscript. AM participated in the experimental design and manuscript preparation. SCR participated in the experimental design and production of the draft of the manuscript. All authors have read and approved the final manuscript.

## Supplementary Material

Additional file 1 **Supplementary table S1. Differentially regulated *Salmonella *Typhimurium genes in response to d-limonene**. Fold changes of the genes induced and repressed due the effect of d-limonene on the genome of *S*. Typhimurium is demonstrated in this table. Click here for file
